# Characterisation of novel endogenous geminiviral elements in macadamia

**DOI:** 10.1186/s12864-021-08174-0

**Published:** 2021-11-27

**Authors:** Mohamed C. M. Zakeel, Andrew D. W. Geering, John E. Thomas, Olufemi A. Akinsanmi

**Affiliations:** The University of Queensland, Queensland Alliance for Agriculture and Food Innovation, Centre for Horticultural Science, GPO Box 267, Brisbane, QLD 4001 Australia

**Keywords:** Rolling circle amplification, Transcriptional activity, Geminivirus, Proteaceae genome, Fossilized viral sequences

## Abstract

**Background:**

The presence of geminivirus sequences in a preliminary analysis of sRNA sequences from the leaves of macadamia trees with abnormal vertical growth (AVG) syndrome was investigated.

**Results:**

A locus of endogenous geminiviral elements (EGE) in the macadamia genome was analysed, and the sequences revealed a high level of deletions and/or partial integrations, thus rendering the EGE transcriptionally inactive. The replication defective EGE in the macadamia genome indicates its inability to be the source of new viral infections and thus cause AVG or any other disease in macadamia. The EGE sequences were detected in two edible *Macadamia* species that constitute commercial cultivars and the wild germplasm of edible and inedible species of *Macadamia*. This strongly suggests that the integration preceded speciation of the genus *Macadamia*. A draft genome of a locus of EGE in *Macadamia* was developed. The findings of this study provide evidence to suggest the endogenization of the geminiviral sequences in the macadamia genome and the ancestral relationship of EGE with *Macadamia* in the Proteaceae family. Random mutations accumulating in the EGE inform that the sequence is evolving.

**Conclusions:**

The EGE in *Macadamia* is inactive and thus not a direct cause of any diseases or syndromes including AVG in macadamia. The insertion of the EGE in the macadamia genome preceded speciation of the genus *Macadamia*.

**Supplementary Information:**

The online version contains supplementary material available at 10.1186/s12864-021-08174-0.

## Introduction

Viral DNA that inserts in the nuclear genome of its host organism germline cells and therefore is transmitted between host generations like a normal cellular gene is referred to as an endogenous viral element (EVE). EVEs are very common in the plant kingdom, and often derive from DNA viruses in either the *Caulimoviridae* or *Geminiviridae* families [1, 2]. As EVEs are essentially molecular fossils of viruses that may have existed millions of years ago, they provide valuable insights into virus evolution [3, 4]. EVEs also likely play a major part in plant evolution by contributing novel protein-coding genes or exons [5, 6], and may also help shape the transcriptome by being sources of novel promoter regulatory elements or non-coding small RNAs [7]. In some cases, the EVEs represent entire viral genomes, retain replication-competency and can be the origin of new infections [8–11].

Macadamia, an important nut-bearing tree that originates from Australia but is now commercially cultivated in subtropical regions throughout the world, is typical of many plants by having EVEs. At least three types of EVE are present in *Macadamia integrifolia*, two of which are derivatives of ancestral petu- and florendoviruses from the family *Caulimoviridae* [1], and a third that is representative of an unassigned genus in the family *Geminiviridae* [2]. All EVEs were identified using software pipelines to interrogate plant genome databases for the presence of highly conserved viral genes such as the replication-associated (Rep) protein of the *Geminiviridae*. The *Rep* gene in the macadamia genome is flanked by sequences encoding other conserved viral domains, which suggests that a complete or near complete viral genome had been integrated in the macadamia genome. In a phylogenetic analysis, Sharma et al. [2] placed the macadamia *Rep* gene sequence in a clade of endogenous geminiviral elements (EGEs) from *Citrus*, *Coffea* and *Camelia* spp., which together are basal to the genus *Begomovirus*. As there are currently no publicly available transcriptome databases for macadamia, Sharma et al. [2] were not able to judge whether the macadamia EGE was transcriptionally active using a bioinformatics approach alone.

In Australia, macadamia is afflicted by a disorder known as abnormal vertical growth (AVG) syndrome. The symptoms of AVG are vigorous upright growth, a reduction in the production of lateral shoots and far fewer flowers, leading to serious yield loss [12]. The cause of AVG has remained elusive despite at least two decades of research on the problem. Analyses of the spatiotemporal dynamics of the AVG epidemic over a 10-year period suggest slow spread over very short distances, consistent with a biotic cause for the disease [13]. It has been suggested that AVG is caused by a vascular-limited organism that has the capacity to modulate plant hormone metabolism [13].

In this study, we hypothesized that the EGE in macadamia is the cause of AVG. To test this hypothesis, a locus containing a putative full-length viral genome sequence was characterized and the replication-competency of this sequence examined by searching for mutations that could disrupt the open reading frames (ORFs). Other tests of replication competency were done by testing for RNA transcripts and examining whether single-stranded, circular DNA molecules that corresponded to the encapsidated form of the virus genome were present in the plant.

## Results

### Characterisation of EGEs in the genome of *M. integrifolia*

A tBLASTN search of the most recent chromosome scale assembly of the macadamia genome (NCBI BioProject: PRJNA593881), using the tomato leaf curl virus (TLCV) Rep protein sequence as the query, revealed highly significant matches (E values < 5 × 10^− 40^) to loci on four pseudochromosomes (4, 5, 9 and 13) and on two other orphan scaffolds (134 and 2940). Only three matches appeared to have full-length *Rep* gene sequences based on query coverage of greater than 90%. All coding sequences were interrupted by mutations that led to the insertion of at least one internal stop codon within the predicted ORF. Pseudochromosome 9 was of particular interest as it had a 3751 bp stretch of sequence that contained one complete and two partial *Rep* genes, suggesting a multimeric insertion of geminiviral sequence.

**Fig. 1:**
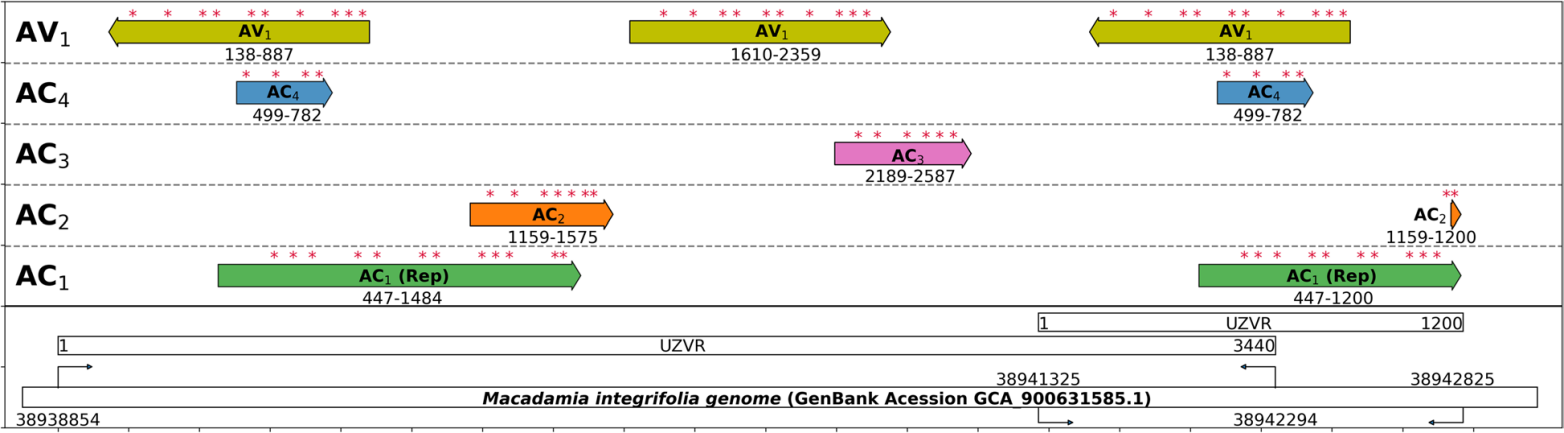
Endogenized geminivirus locus in *Macadamia integrifolia* genome. Asterisk marks indicate mutations that have caused disruptions to the open reading frames. AC_1_, AC_2_, AC_3_, AC_4_ and AV_1_ indicate genes that encode replication-associated protein (Rep), a translational activator protein (TrAP), a replication enhancer protein (REn), a replication enhancer protein and coat protein, respectively

PCR primer walking and Sanger sequencing were done to validate the EGE sequence extracted from the genome assembly of *M. integrifolia* and both sequences were identical. The sequence that was obtained by Sanger sequencing has been deposited in GenBank under accession MZ474517. Closer inspection of this locus revealed a monomeric unit of 3440 bp, which was partially duplicated immediately downstream (Fig. 1). By BLASTX comparison with the various begomovirus proteins, Rep, AC2, AC3 and AC4 and two AV1 genes in opposite directions were identified, all of which contained mutations that interrupted the ORFs (Fig. 1). At the cut off level of mutations tolerated to find gene sequences, AC3 and AC4 genes were not found internally in the second and first copies of AV1 sequences, respectively (Fig. 1). A conserved geminivirus origin of replication (ori) with the sequence of TAATATTAC was found. In addition, an ori sequence of TGAGATTCC, which had the second base mutated compared to a *Becurtovirus* ori, was identified 262 nt upstream of the previous ori sequence.

**Fig. 2:**
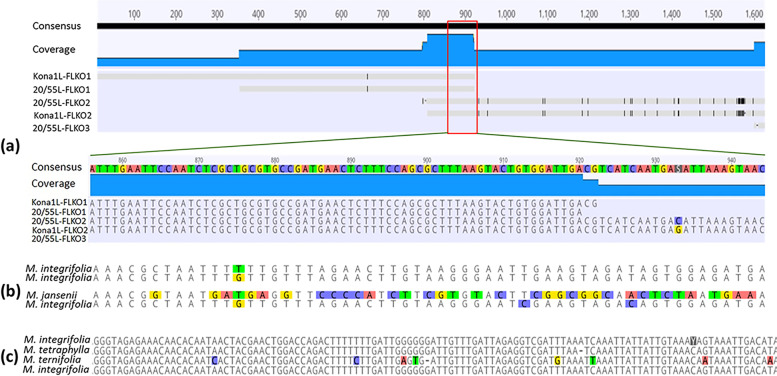
Assemblage of endogenous geminiviral element (EGE) sequence in macadamia genome. **a** A part of EGE in *M. integrifolia* genome **b** & **c** Alignments of two different segments of EGE in different *Macadamia* species

**Fig. 3:**
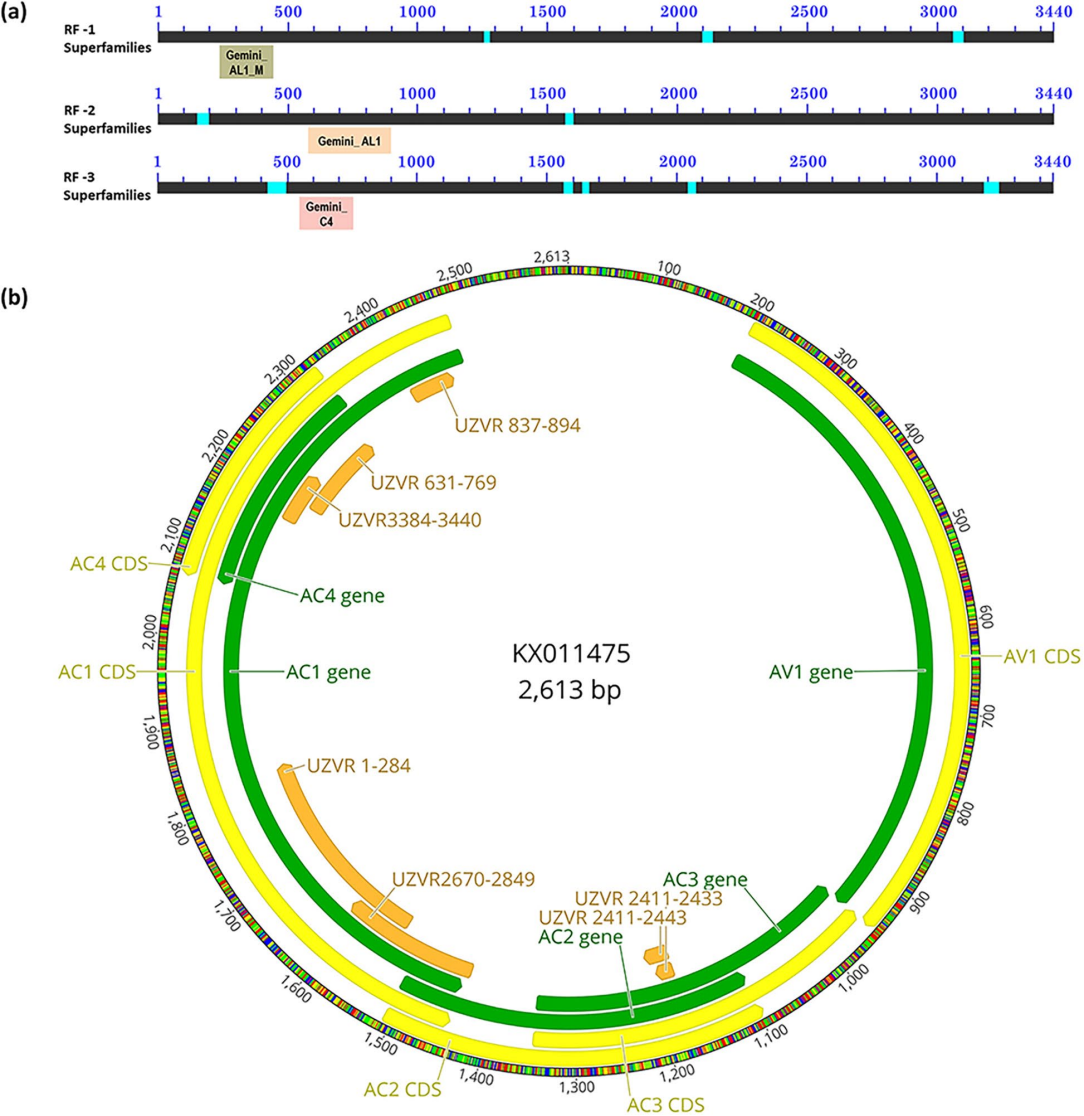
Fragments of putative conserved protein domains predicted in assembled contig of endogenous geminiviral element (EGE) (**a**) UZVR_3440 bp present in macadamia genome, and (**b**) genome map of a representative begomovirus, *common bean severe mosaic virus* (KX011475), with matching fragments of the EGE in macadamia. Sandy brown, limestone and light coral boxes indicate geminivirus Rep catalytic domain (AL1), Rep protein central domain (AL1_M) and C4 protein, respectively. The protein domains were predicted in different reading frames (RF). Compositionally biased regions of the RF (light blue bars) were not used in domain database search. Gene (green) and coding sequences (CDS, yellow) of the representative viral genome and the matching sequences of EGE (orange) are marked in the map

The final assemblage of the macadamia EGE (UZVR_3,440 bp) produced using overlapping sequences obtained from *Macadamia integrifolia* (Fig. 2a; GenBank accession MZ474517), predicted three putative partial conserved domains, including geminivirus Rep catalytic domain, geminivirus Rep protein central domain and geminivirus C4 protein (Fig. 3). A geminivirus Rep protein central domain (AL1_M, 252–440 bp), Rep catalytic domain (AL1, 563–895 bp) and an N terminus region of C4 or AC4 protein (586–756 bp) were predicted in reading frames 1, 2 and 3 of the final EGE assemblage, respectively (Fig. 3a). The genome organization of the macadamia EGE was similar to monopartite begomoviruses. Although the sequences of above domains were highly conserved in macadamia EGE, most of the other domains of begomovirus were not detected or showed substantial level of mutations.

Many begomoviruses carry betasatellite DNA components, which modulate symptoms and enhance viral DNA accumulation but are nonessential for viral replication. A protein encoded by a complementary sense ORF (βC1) on the betasatellite molecule is the major determinant of pathogenicity [14]. To examine whether the ancestral geminivirus in *M. integrifolia* had a betasatellite, a tBLASTN search of the aforementioned macadamia genome assembly was done using the C1 protein sequence of a tomato begomovirus betasatellite DNA (GenBank Accession NP_859031) as the query but no significant hits were obtained.

### Transcriptional activity and replication competency of EGE in macadamia

**Fig. 4:**
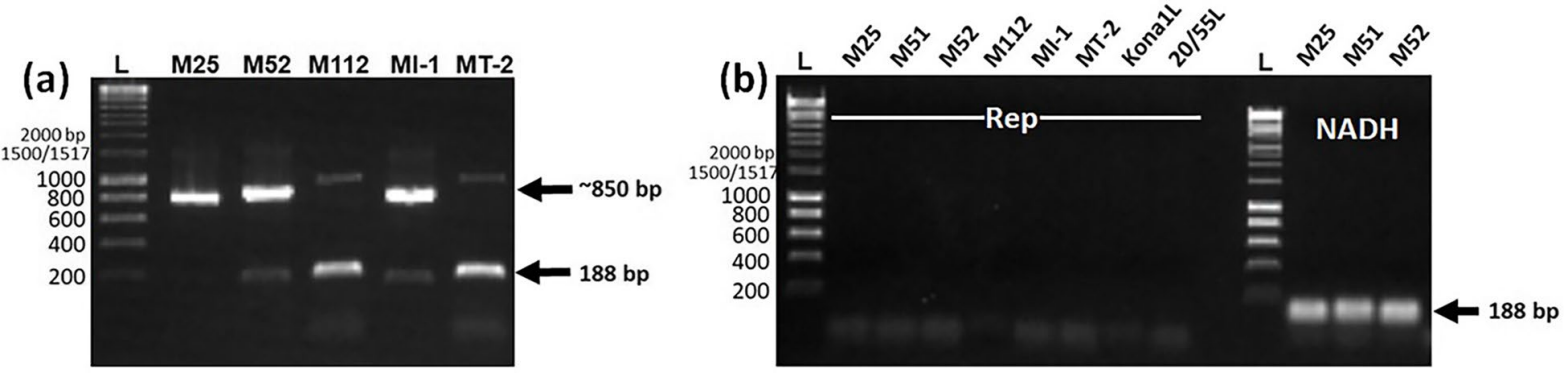
PCR amplicons of DNA and cDNA to evaluate the transcriptional activity of Rep protein of EGE in macadamia. **a** PCR products of total nucleic acid with *NADH* primer pair (Nad2.1a and Nad2.2b) in 1% agarose gel. **b** PCR amplicons, which were obtained from cDNA with *Rep* and *NADH* primers, in a 1% agarose gel

While sequence decay was observed at the EGE locus that was examined, it may still be possible that an infective geminivirus genome could be released through several DNA recombination events. Alternatively, the plant may be infected with a descendant of the virus that became endogenized. Two indicators of geminivirus infection are the presence of RNA transcripts and of circular DNA molecules and experiments were done to address each of these points. The search for transcription of the virus genome, with RT-PCR assays targeting the conserved *Rep* gene using RNA extracts from eight samples including both AVG and healthy trees, revealed no amplification. The primer-binding sites were located in exons that were separated by an intron, allowing amplicons from carryover DNA to be separated by size from those obtained with the spliced RNA transcript. Accordingly, two amplicons of approximately 850 bp (DNA) and 188 bp (mRNA) were obtained with NADH primer pair using a total nucleic extract as template (Fig. 4; Supplementary Fig. 1). The results obtained showed that there were no detectable levels of *Rep* gene transcript in any sample, nor problems with DNA contamination of the RNA extracts.

Finally, we were unable to detect circular forms of the geminiviral DNA using a sequence non-specific amplification assay (TempliPhi), which also suggested that there were no active infections (Supplementary Fig. 2).

### Presence of EGE orthologues in the four *Macadamia* species in Proteaceae

**Fig. 5:**
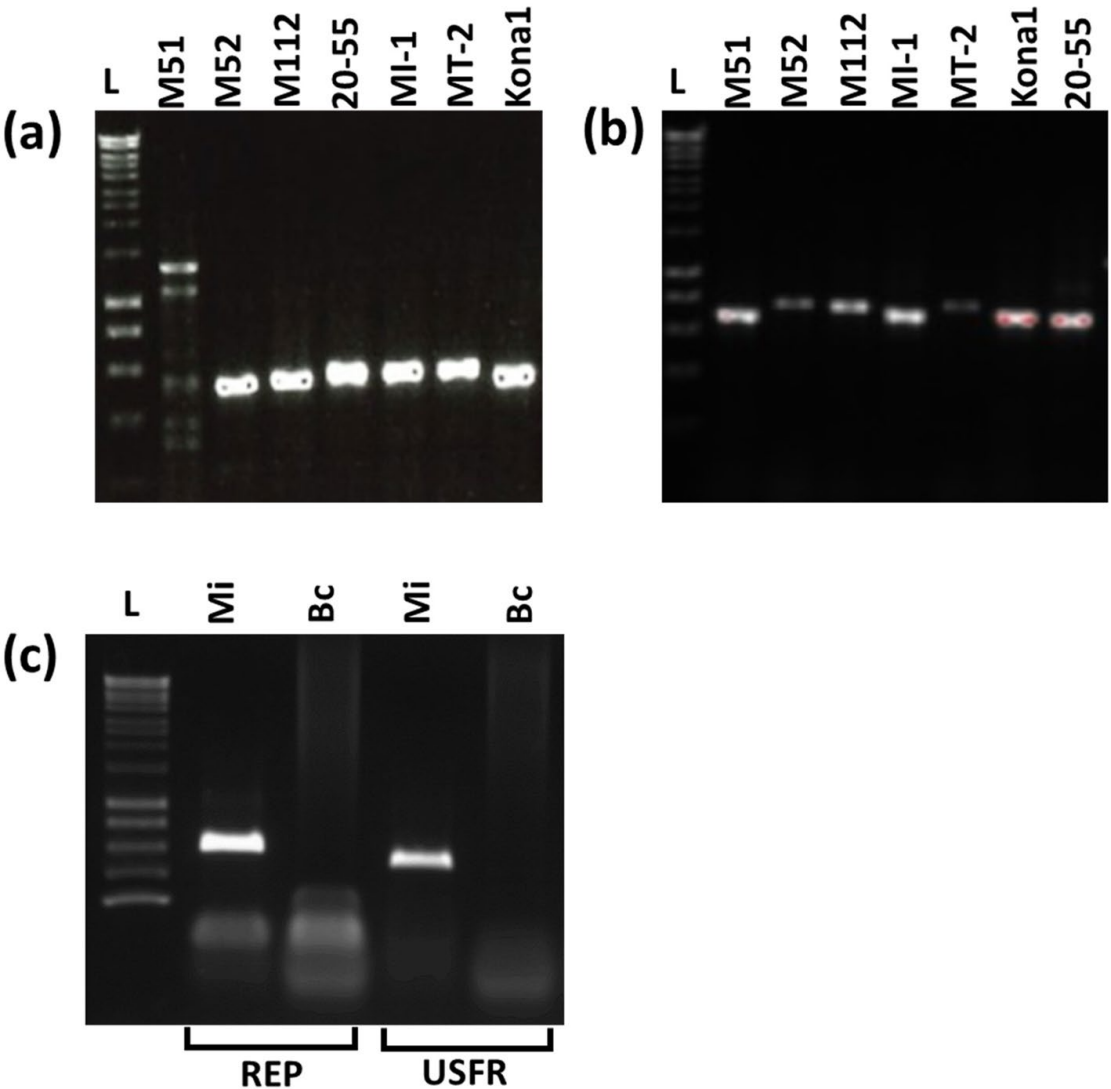
PCR amplification revealed the presence of (**a**) upstream and (**b**) downstream flanking regions of EGE in different *Macadamia* species (M51: *M. jansenii*; M52: *M. ternifolia*; MT-2: *M. tetraphylla*; M112, MI-1, Kona1 and 20–55 represent *M. integrifolia*), and (**c**) the presence or absence of Rep and upstream flanking regions in *M. integrifolia* and *Buckinghamia celsissima* genomes. L: Bioline 1 kb hyperLadder

To search for orthologues of 3.4 kb EGE locus in the four *Macadamia* species, PCR assays were designed to amplify the junction between plant and viral sequence at either end of the locus. The expected amplicons for both junction sequences were present in all the macadamia species (Fig. 5a, b; Supplementary Fig. 1). Furthermore, two additional amplicons representing the upstream junction sequence were obtained for *M. jansenii* (M51) (Fig. 5a; Supplementary Fig. 1), indicating a more complicated integration pattern in this plant species (Fig. 5a; Supplementary Fig. 1). When the analysis was extended to a more distantly related member of the Proteaceae, *Buckinghamia celssia* (Ivory curl), evidence was obtained for the absence of the locus using the PCR for the upstream junction sequence, as well using a PCR for the *Rep* gene sequence (Fig. 5c; Supplementary Fig. 1). Compared to the EGE sequences of *M. integrifolia* and *M. tetraphylla*, partial EGE sequences (~ 600 bp) developed in *M. jansenii* and *M. ternifolia* showed 60 and 10% mutations, respectively (Fig. 2b, c). The number of mutations were likely to be proportional to genetic relatedness of the *Macadamia* species.

## Discussion

In this study we have characterized the EGE in *M. integrifolia* that was first identified by Sharma et al. [2], and provided compelling evidence that it is replication-defective and therefore very unlikely to be a direct cause of AVG. The EGE in *Macadamia* appears to be accumulating random mutations, suggesting that there is not strong selection pressure for maintenance of the viral ORFs. Our study using RT-PCR confirmed the transcriptional inactivity of *Rep* sequence of EGE in macadamia. Further, a draft genome of the EGE in macadamia was developed.

Our results suggest that the insertion of the EGE in the macadamia genome preceded speciation of the genus *Macadamia*. Complex evolutionary associations between viruses and their hosts have been revealed by endogenous viral elements [15]. Most endogenous viral elements are functionally defective in hosts, but some endogenous viral elements have retained their role in some hosts or may acquire certain functions, advantageous to the host [16, 17]. The fact that the EGE has persisted through several host radiation events without being expunged suggests that it is serving some useful function to the plant. Australia, the place of origin of *Macadamia*, has ancient soils that are deficient in both nitrogen and phosphorous, the two building blocks of DNA. Hence, there presumably has been strong selection pressure against the development of genome obesity in *Macadamia* through the accumulation of unwanted repetitive DNA elements because of the extra nutritional demands placed on the plant [18]. The insertion of either an endogenous viral element or a retrotransposon into or near a gene can alter the activity and function of that gene by a variety of mechanisms such as by contributing novel transcriptional regulatory motifs or by inducing transcriptional silencing [19–21]. Insertion of the EGE could therefore have enabled rapid change in the metabolism and therefore phenotype of the plant in the face of a new environmental stress. The EGE could also confer resistance to the cognate exogenous virus if it should still exist [17].

Mutations and recombination events in the EGE in macadamia may have led to the reduction in the number of conserved domains found in the *Macadamia* EGE. Transcriptionally inactive sequences of EGE in macadamia also indicate that the sequences are methylated or decayed. Mutational decay is the major cause for endogenous viral elements to be defective and for reduction in the number of conserved domains [4, 22].

The absence of circular forms of geminiviral elements indicates that the EGE is not activated to be the origin of new viral infections. Endogenized viral sequences may be activated under stress condition, resulting in disease in the host. For example, stress factors such as micropropagation by tissue culture was found to trigger the activation of endogenous banana streak viruses in *Musa* cultivars [23]. The heavily mutated ORF of the *Rep* sequence of the EGE confirmed that the sequence is replication defective and thus not activated to cause any diseases such as AVG. A functional *Rep* protein is required for the replication of geminiviral DNA [24]. In particular, motifs 1–3 and helices 1 and 2 in the N-terminal domain of the Rep protein of geminivirus are crucial for the replication of *Tomato golden mosaic virus* DNA [25]. Heavily mutated motifs and helices of the *Rep* sequence of the EGE is another indication of its transcriptional inactivity.

The *Macadamia* EGE falls within ‘clade 2’ of unassigned EGEs, a clade that also includes EGEs from *Camelia*, *Coffea*, *Tectona*, *Diospyros* and *Argania* [2]. Collectively these genera are palaeotropical in distribution, making it difficult to determine the precise origin of this group of viruses. However, at least two of the genera of host plants, *Macadamia* and *Coffea* which form a separate sub-clade [2], have clear Gondwanan links. *Coffea* has centres of origin in central Africa and Madagascar, whereas *Macadamia* is uniquely Australian and belongs to the Proteacee, which almost exclusively has a Southern Hemisphere distribution. It was evident that the genome organization of *Macadamia* EGE seems to be unique as virus and complementary sense genes are overlapping, which is not observed in any extant geminiviruses. However, the phylogenetic analysis of *Rep* sequence of the EGE with other geminiviruses have shown that the ‘clade 2’ EGEs are basal to the genus *Begomovirus* [2], which also have centres of diversity in South America and India, and therefore it would seem likely that this whole group of viruses evolved in Gondwana.

## Conclusions

The EGE in *Macadamia* is replication-defective and thus not the origin of new viral infection. Therefore, it is very unlikely that EGE in *Macadamia* is a direct cause of AVG. The insertion of the EGE in the macadamia genome preceded speciation of the genus *Macadamia* and it is likely that the EGE is playing some useful role in the plant.

## Materials and methods

### Plant materials and nucleic acid extractions

Leaf samples were collected from commercial macadamia trees with the permission of the owners at each private farm in Bundaberg and from the wild accessions in ex-situ germplasm conservation site in Queensland with the permission of the lead program manager (Professor Bruce Topp, The University of Queensland, National Macadamia Breeding and Conservation). We also collected leaf samples from the common ornamental tree *B. celsissima*, which is a close relative of macadamia and commercially available for ornamental purpose in Australia. All relevant institutional, national, and international guidelines, legislation and protocols were followed for the collection of the samples [26]. The identity of all plant materials was confirmed by O. A. Akinsanmi, The University of Queensland. Sampling methods used in this study are described in Zakeel et al. [27]. Sources and specimens for all plant materials used in this study are accessible in public collection in Australia. Total nucleic acids and pure RNA were extracted from the lamina and midrib of leaf samples using the CTAB method of Rogers and Bendich [28], and the TRIzol reagent as per the manufacturer’s instructions, respectively. RNA was quantified using a μDrop Plate (Thermo Scientific).

### Characterisation of the EGE in the macadamia genome

EGEs were identified in the *M. integrifolia* genome (GenBank Accession GCA_900631585.1) [29] by doing a tBLASTN search using the Rep protein of TLCV (NCBI protein database accession P36279) as the query sequence. Previous GenBank accession (UZVR00000000; NCBI BioProject: PRJEB13765) of *M. integrifolia* genome has same sequence for this locus. The endogenous geminiviral sequences were then extended by comparing different loci containing identical or near-identical sequences by doing pairwise sequence alignments using BLASTN. To derive a consensus sequence (Supplementary Data 1), fragments of sequence from different loci were assembled using the contig assembly algorithm in Geneious v. 10.2.5 (Biomatters Ltd) operated using default settings.

Using Mac_Gem F2 and Mac_Gem R2 primer pair (Table 1), a PCR was carried out with total nucleic acids extracted from AVG-symptomatic and healthy macadamia trees and the amplicons were sequenced to verify the presence of the viral locus in the NGS assembly. Each PCR was done in a 25 μl reaction containing 2 μl of total nucleic acid, 1 μl of 10 × Mango *Taq* reaction buffer (Bioline), 4 mM MgCl_2_, 200 μM of each dNTP, 500 nM of each primer, 2% DMSO, 0.04 μg/μl BSA, 1 unit of Mango*Taq* DNA polymerase (Bioline) and nuclease-free water to the final volume. Thermocycling conditions were an initial denaturation at 95 °C for 5 min, followed by 35 cycles of denaturation at 95 °C for 30 s, annealing at 46 °C for 30 s and extension at 72 °C for 30 s, with a final extension step at 72 °C for 5 min. Amplicons were separated in a 1% agarose gel in 0.5 × TBE, stained in ethidium bromide for 10 min and visualized on a UV transilluminator. PCR products with an expected size of 870 bp were sequenced at the Macrogen Inc. (Seoul, South Korea).

**Table.1:** Details of primers used in this study

Primer name	Sequence (5′–3′)	Target	Annealing temperature (°C)	Source
Mac_Gem_RepF1	CTGGACTTTGATGCTCGGTGGCATAG	*Rep*	58	Zakeel et al. [27]
Mac_Gem_RepR1	CAAGGAACCAAAACCACCATGCTG			
Nad2.1a	GGACTCCTGACGTATACGAAGGA	*NADH*	56	Thompson et al. [30]; Murray Sharman (*Pers. Comm*.)
Nad2.2b	AGCAATGAGATTCCCCAATATCAT			
Mac_Gem F2	GATTTAAAGAACCACAATGAT	*Rep*	46	Akinsanmi [26]
Mac_Gem R2	CTTAATGCATCATTTACTGAAC			

To validate the whole genome shotgun sequence assemblies of loci containing EGEs, PCR products that overlapped each other by 150–200 bp were generated using the primers listed in Table 2. Each 50 μl PCR mix contained 1 × Mango*Taq* reaction buffer (Bioline), 4 mM MgCl_2_, 200 μM of dNTPs, 200 nM of each primer, 2% DMSO, 0.04 μg/μl BSA, 2 units of Mango*Taq* DNA polymerase (Bioline), 2 μl total nucleic acid template (≤ 10 ng/μl) and nuclease-free water to the final volume. Using the same thermocycling conditions described above. PCR products were purified using a QIAGEN PCR purification or gel purification kits according to the manufacturer’s instructions and sequenced at the Macrogen Inc., Seoul, South Korea, using the Sanger sequencing method. Sequences were trimmed, processed and assembled in Geneious.

**Table 2:** Primers for flanking regions and overlapping fragments of macadamia associated geminiviral genome

Accession No.	Primer name	Sequence	Annealing temperature (°C)	Target
UZVR01000870	USFR_F	CACCAGCTCAAAGTATCTCC	52 (USFP)^a^	Up and downstream flanking region
	USFR_R	ATGTAAGCCTTCCCTCTCGA		
	DSFR_F1	GGTTACCATCTGACATGCTCAAGGCT	61 (DSFP)^a^	
	DSFR_R1	TGCTGGAACTGTGGTCTGATTTCAGTCC		
	UZVR1_F3	GGTCGTACTTTCGATCTCTGATCTGAGG	56	Macadamia associated EGE assemblage 1
	UZVR1_R3	GGGTGGAATCCCAAGTATTGTCTTATGC		
	UZVR_2F	CCACATTCTGTATCGTCCAT	53	
	UZVR_2R	TATCCTAAATGTCCACTTCC		
	UZVR_3F	CTCCTTTGGAAGTGGACATTTA	55	
	UZVR_3R	ATAAGAGAGTCCTTATCCTCC		
	UZVR_4F	GTTGCGGTTATTGAGTCGAA	54	
	UZVR_4R	GAGCATCAAAGTCCAGTATC		
	UZVR_5F	GTGACACCGACATTCGAACT	54	
	UZVR_5R	AGTTGCCGCAGAAGTACACG		
	UZVR_6F	TACTTCTGCGGCAACTCTGATG	53	
	UZVR_6R	GTCGAGTCTGTCACTCCTTC		
FLKO01046132	FLKO_1F	TTAGGGCATGGGTGTAAAATC	60	Macadamia associated EGE assemblage 2
	FLKO_1R	AAGAACCACAATGATGACTG		
	FLKO_2F	GATAATCCTAGACCTGGATTGC	56	
	FLKO_2R	ATATTCCATATGCCGCGTTC		
	FLKO_3F	CTGAACAGAGCTTGAATCAG	55	
	FLKO_3R	GAGAGTCCTTATCCTCCATG		

^a^*USFP* Upstream flanking primers, *DSFP* Downstream flanking primers

### RT-PCR

To investigate whether the EGE in macadamia is transcribed, reverse transcription PCR was done using the primers listed in Table 1. As templates, total RNA extracts from leaves of eight trees, including those from AVG and non-AVG trees, were used (Table 3). As an internal control, RT-PCRs were done to detect the single copy mitochondrial gene *NADH* using the primers of Thompson et al. [30], except the forward primer primer was modified so that it no longer spanned exon junctions and amplified both the DNA copy of the gene and its spliced RNA transcript (Murray Sharman, *Pers. Comm.*; Table 1). Amplicons arising from contaminating DNA in the RNA extract were able to be distinguished by size, as the DNA contains an intron.

**Table 3:** Plant samples used in this study

Sample no.	***Macadamia*** species	Source	AVG symptoms
M25	*M. integrifolia*	Cultivar HAES 344	AVG
20/55 L	*M. integrifolia*	Cultivar HAES 344	AVG
Kona1L	*M. integrifolia*	Cultivar HAES 344	Non-AVG
M112	*M. integrifolia*	Wild germplasm	Non-AVG
MI-1	*M. integrifolia*	Wild germplasm	Non-AVG
MT-2	*M. tetraphylla*	Wild germplasm	Non-AVG
M51	*M. jansenii*	Wild germplasm	Non-AVG
M52	*M. ternifolia*	Wild germplasm	Non-AVG

To synthesize cDNA, 2 μl of 10 μM reverse primer (either Mac_Gem_RepR1 or Nad2.2b), 2.5 μl nuclease-free water and 3 μl freshly prepared RNA were incubated at 80 °C for 10 min, followed by snap-chilling on ice. An aliquot of 4 μl of master mix containing 2.5 × first strand buffer, 25 mM DTT, 1.25 mM dNTPs, 50 units SuperScript III (Invitrogen) reverse transcriptase and 10 units RNase out was then added to the tube. Reverse transcription was done at 55 °C for 45 min, then the enzyme denatured at 70 °C for 10 min. PCR was done in a 25 μl reaction volume containing 1 μl of cDNA, 1 × Mango*Taq* reaction buffer (Bioline), 1.75 mM MgCl_2_, 0.2 mM dNTPs, 0.2 μM of each primer, 1 unit Mango*Taq* polymerase (Bioline) and nuclease-free water to make up the volume. Thermocycling conditions were an initial denaturation step at 95 °C for 5 min, 35 cycles of denaturation at 95 °C for 30 s, primer annealing at the respective temperature (Table 1) for 30 s and extension at 72 °C for 30 s, and a final extension step of 72 °C for 5 min. Separate RT-PCRs were done to test for EGE and NADH RNA transcripts. The PCR products were electrophoresed in a 1% agarose gel, stained in ethidium bromide and visualized on a UV transilluminator.

### Rolling circle amplification

To amplify circular DNA molecules, rolling circle amplification (RCA) was done using a TempliPhi 100 DNA amplification kit (GE Healthcare, Buckinghamshire, United Kingdom) as per the manufacturer’s protocol but with the reaction mixture spiked with 1 μl each of the primers Mac_Gem F2 and Mac_Gem R2 at 10 μM concentration to improve the sensitivity of detection. The plasmid pUC19 was used as a positive control. Three μl of RCA product was then digested with 10 units of either *Bam*HI, *Eco*RI or *Hin*dIII (New England BioLabs Inc.) at 37 °C for 24 h in a 10 μl reaction mixture containing 1 × CutSmart® buffer (BioLabs Inc., New England). The restriction enzymes were selected based on the presence of recognition sites in the EGE (*Eco*RI and *Hin*dIII) or TLCV (*Bam*HI) which was initially used to identify EGE in macadamia genome. Digested products were separated in a 1% agarose gel, stained in ethidium bromide and visualized under UV illumination.

### Search for EGE orthologues in the four *Macadamia* species

To search for orthologues of the EGE in the different *Macadamia* spp., PCR primers were designed to anneal on either side of the junction between plant and geminiviral sequence (Table 2). Each 25 μl assay mixture contained 1 × Mango*Taq* reaction buffer, 4 mM MgCl_2_, 200 μM of dNTPs, 200 nM of each primer, 2% DMSO, 0.04 μg/μl BSA, 1 unit of Mango*Taq* DNA polymerase, 1 μl of total nucleic acid extract (≤ 10 ng/μl) and nuclease-free water to the final volume. Thermocycling conditions were as described above. PCR amplicons were electrophoresed in a 1% agarose gel and visualised under UV. To check if the EGE sequences are present in Proteaceae that originated from the same region as *Macadamia* (*M. integrifolia, M. jansenii* and *M. ternifolia*) in Queensland, ivory curl (*B. celsissima*) that is endemic to the wet tropics rainforest areas of northeastern Queensland, was selected. PCR reactions were carried out with ivory curl genomic DNA samples to amplify *Rep* sequence and upstream flaking regions (Table 1).

## Supplementary Information

**Additional file 1: Supplementary Data 1.** Consensus sequence of endogenous geminiviral elements of macadamia initially developed by computational approach and pairwise sequence alignment and contig assembly methods.

**Additional file 2: Supplementary Figure 1.** Full length gel images of Figs. 4a, b and 5a, b and c included in this article.

**Additional file 3: Supplementary Figure 2.** Detection of circular forms of geminiviral DNA using the TempliPhi assay, a sequence non-specific amplification method.

## Data Availability

The original contributions presented in the study are included in the article or supplementary material. The draft genome sequence can be accessed from NCBI (GenBank accession MZ474517) when it becomes publicly available. *Macadamia integrifolia* genome sequence initially analysed in this study can be accessed from NCBI (GenBank Accession GCA_900631585.1). Other data generated and/or analysed during the current study are available from the data access/ethics committee of The University of Queensland (UQ), but restrictions apply to data availability. Data are however available from the authors upon reasonable request and with permission of UQ for researchers who meet the criteria for access to confidential data. Raw sanger sequencing data can be accessed from 10.14264/f801edd. Further inquiries can be directed to the corresponding authors.

## Abbreviations

AVG: Abnormal vertical growth
EGE: Endogenous geminiviral elements
EVE: Endogenous viral element
ORF: Open reading frames
RCA: Rolling circle amplification
TLCV: Tomato leaf curl virus

## References

[1] Diop SI, Geering AD, Alfama-Depauw F, Loaec M, Teycheney P-Y, Maumus F. Tracheophyte genomes keep track of the deep evolution of the *Caulimoviridae*. Sci Rep. 2018;8(1):572. 10.1038/s41598-017-16399-x. 10.1038/s41598-017-16399-x PMC5766536 29330451

[2] Sharma V, Lefeuvre P, Roumagnac P, Filloux D, Teycheney P-Y, Martin DP, et al. Large-scale survey reveals pervasiveness and potential function of endogenous geminiviral sequences in plants. Virus Evol. 2020;6(veaa071). 10.1093/ve/veaa071. 10.1093/ve/veaa071 PMC7758297 33391820

[3] Aiewsakun P, Katzourakis A. Endogenous viruses: connecting recent and ancient viral evolution. Virology. 2015;479-480:26–37. 10.1016/j.virol.2015.02.011. 10.1016/j.virol.2015.02.011 25771486

[4] Geering AD, Maumus F, Copetti D, Choisne N, Zwickl DJ, Zytnicki M, et al. Endogenous florendoviruses are major components of plant genomes and hallmarks of virus evolution. Nat Commun. 2014;5(1):5269. 10.1038/ncomms6269. 10.1038/ncomms6269 PMC4241990 25381880

[5] Carrasco JL, Sánchez-Navarro JA, Elena SF. Exploring the role of cellular homologous of the 30K-superfamily of plant virus movement proteins. Virus Res. 2019;262:54–61. 10.1016/j.virusres.2018.02.015. 10.1016/j.virusres.2018.02.015 29475053

[6] Liu H, Fu Y, Jiang D, Li G, Xie J, Cheng J, et al. Widespread horizontal gene transfer from double-stranded RNA viruses to eukaryotic nuclear genomes. J Virol. 2010;84(22):11876–87. 10.1128/JVI.00955-10. 10.1128/JVI.00955-10 PMC2977895 20810725

[7] Grandi N, Tramontano E. Human endogenous retroviruses are ancient acquired elements still shaping innate immune responses. Fron Immunol. 2018;9(2039). 10.3389/fimmu.2018.02039. 10.3389/fimmu.2018.02039 PMC6139349 30250470

[8] Chabannes M, Baurens F-C, Duroy P-O, Bocs S, Vernerey M-S, Rodier-Goud M, et al. Three infectious viral species lying in wait in the banana genome. J Virol. 2013;87(15):8624–37. 10.1128/JVI.00899-13. 10.1128/JVI.00899-13 PMC3719817 23720724

[9] Lockhart BE, Menke J, Dahal G, Olszewski NE (2000). Characterization and genomic analysis of tobacco vein clearing virus, a plant pararetrovirus that is transmitted vertically and related to sequences integrated in the host genome. J Gen Virol.

[10] Ndowora T, Dahal G, LaFleur D, Harper G, Hull R, Olszewski NE, et al. Evidence that badnavirus infection in *Musa* can originate from integrated pararetroviral sequences. Virology. 1999;255(2):214–20. 10.1006/viro.1998.9582. 10.1006/viro.1998.9582 10069946

[11] Richert-Pöggeler KR, Noreen F, Schwarzacher T, Harper G, Hohn T. Induction of infectious petunia vein clearing (pararetro) virus from endogenous provirus in petunia. EMBO J. 2003;22(18):4836–45. 10.1093/emboj/cdg443. 10.1093/emboj/cdg443 PMC212712 12970195

[12] O'Farrell P, Le Lagadec D, Searle C. 'Abnormal vertical growth': a disorder threatening the viability of the Australian macadamia industry. Acta Hortic. 2016;1109:143–50. 10.17660/ActaHortic.2016.1109.23.

[13] Zakeel MCM, Geering ADW, Akinsanmi OA. Spatiotemporal spread of abnormal vertical growth of macadamia in Australia informs epidemiology. Phytopathology. 2020;110(7):1294–304. 10.1094/PHYTO-10-19-0396-R. 10.1094/PHYTO-10-19-0396-R 32223641

[14] Saeed M, Behjatnia SA, Mansoor S, Zafar Y, Hasnain S, Rezaian MA. A single complementary-sense transcript of a geminiviral DNA β satellite is determinant of pathogenicity. Mol Plant-Microbe Interact. 2005;18(1):7–14. 10.1094/MPMI-18-0007. 10.1094/MPMI-18-0007 15672813

[15] Katzourakis A, Gifford RJ. Endogenous viral elements in animal genomes. PLoS Genet. 2010;6(11):e1001191. 10.1371/journal.pgen.1001191. 10.1371/journal.pgen.1001191 PMC2987831 21124940

[16] Holmes EC. The evolution of endogenous viral elements. Cell Host Microbe. 2011;10(4):368–77. 10.1016/j.chom.2011.09.002. 10.1016/j.chom.2011.09.002 PMC7172163 22018237

[17] Mette M, Kanno T, Aufsatz W, Jakowitsch J, Van der Winden J, Matzke M, et al. Endogenous viral sequences and their potential contribution to heritable virus resistance in plants. EMBO J. 2002;21(3):461–9. 10.1093/emboj/21.3.461. 10.1093/emboj/21.3.461 PMC125834 11823438

[18] Leitch A, Leitch I. Genomic plasticity and the diversity of polyploid plants. Science. 2008;320(5875):481–3. 10.1126/science.1153585. 10.1126/science.1153585 18436776

[19] Kashino-Fujii M, Yokosho K, Yamaji N, Yamane M, Saisho D, Sato K, et al. Retrotransposon insertion and DNA methylation regulate aluminum tolerance in European barley accessions. Plant Physiol. 2018;178(2):716–27. 10.1104/pp.18.00651. 10.1104/pp.18.00651 PMC6181041 30093528

[20] Wang L, Norris ET, Jordan I. Human retrotransposon insertion polymorphisms are associated with health and disease via gene regulatory phenotypes. Front Microbiol. 2017;8:1418. 10.3389/fmicb.2017.01418. 10.3389/fmicb.2017.01418 PMC5539088 28824558

[21] Hsu C-C, Su C-J, Jeng M-F, Chen W-H, Chen H-H. A *HORT1* retrotransposon insertion in the PeMYB11 promoter causes harlequin/black flowers in Phalaenopsis orchids. Plant Physiol. 2019;180(3):1535–48. 10.1104/pp.19.00205. 10.1104/pp.19.00205 PMC6752922 31088902

[22] Patzke S, Lindeskog M, Munthe E, Aasheim H-C. Characterization of a novel human endogenous retrovirus, HERV-H/F, expressed in human leukemia cell lines. Virology. 2002;303(1):164–73. 10.1006/viro.2002.1615. 10.1006/viro.2002.1615 12482668

[23] Cote FX, Galzi S, Folliot M, Lamagnere Y, Teycheney PY, Iskra-Caruana ML. Micropropagation by tissue culture triggers differential expression of infectious endogenous Banana streak virus sequences (eBSV) present in the B genome of natural and synthetic interspecific banana plantains. Mol Plant Pathol. 2010;11(1):137–44. 10.1111/j.1364-3703.2009.00583.x. 10.1111/j.1364-3703.2009.00583.x PMC6640322 20078782

[24] Laufs J, Jupin I, David C, Schumacher S, Heyraud-Nitschke F, Gronenborn B. Geminivirus replication: genetic and biochemical characterization of rep protein function, a review. Biochimie. 1995;77(10):765–73. 10.1016/0300-9084(96)88194-6. 10.1016/0300-9084(96)88194-6 8824773

[25] Orozco BM, Hanley-Bowdoin L. Conserved sequence and structural motifs contribute to the DNA binding and cleavage activities of a geminivirus replication protein. J Biol Chem. 1998;273(38):24448–56. 10.1074/jbc.273.38.24448. 10.1074/jbc.273.38.24448 9733736

[26] IUCN: IUCN policy statement on sustainable use of wild living resources. 2000. https://portals.iucn.org/library/efiles/documents/Rep-2000-054.pdf. Accessed 20 October 2021.

[27] Zakeel MC, Akinsanmi OA, Geering AD. Molecular detection of putative pathogens to determine any role in a causal relationship with abnormal vertical growth syndrome of macadamia. Eur J Plant Pathol. 2021. 10.1007/s10658-021-02333-5.

[28] Rogers SO, Bendich AJ, Gelvin SB, Schilperoort RA (1994). Extraction of total cellular DNA from plants, algae and fungi. Plant Molecular Biology Manual.

[29] Nock CJ, Baten A, Barkla BJ, Furtado A, Henry RJ, King GJ. Genome and transcriptome sequencing characterises the gene space of *Macadamia integrifolia* (Proteaceae). BMC Genomics. 2016;17:937. 10.1186/s12864-016-3272-3. 10.1186/s12864-016-3272-3 PMC5114810 27855648

[30] Thompson JR, Wetzel S, Klerks M, Vašková D, Schoen C, Špak J, Jelkmann W (2003). Multiplex RT-PCR detection of four aphid-borne strawberry viruses in *Fragaria* spp. in combination with a plant mRNA specific internal control. J Virol Methods.

