# Supplementary material for: Characterisation of novel endogenous geminiviral elements in macadamia
Source: BMC Genomics. 2021 Nov 27;22:858. doi: 10.1186/s12864-021-08174-0 (PMC8626973; doi:10.1186/s12864-021-08174-0)
Supplement: Supplementary file 1 — Additional file 1: Supplementary Data 1. Consensus sequence of endogenous geminiviral elements of macadamia initially developed by computational approach and pairwise sequence alignment and contig assembly methods. [file 12864_2021_8174_MOESM1_ESM.docx]

**Supplementary data 1** Consensus sequence of endogenous geminiviral elements of macadamia initially developed by computational approach and pairwise sequence alignment and contig assembly methods

>Macadamia_geminivirus_1 2777 bp

TAAAATCTCAGTGCAGACTTCGGCGAGGATGACACTCTTTGGGAACCGAAAGAACAGGAGGGTAGTGTGCGCGGTGAAGAGGAGACGAAAGTTGAGGGTAGAGAAACAACACAATAACTACGAACTGGACCAGACTTTTCTTGATTGGGATGGTTGTTTGATTAGAGGTCGATGTAAATTAAATTATTATTGTAAATAATAATTTGTCAAATAAAAAACTCCTTTATTAATCAAGATCTTGGAAACGTACATGATATTGGTCTACATTGTGATTAAAAAAAAAAACTTAGGTCTTGAAAG

TGCCATTGGGATTACATTGTGTCTCAGAGACTGGACTGAAATCAGACCAGAGTTCCCGCACCATATCTTCATCTTCTGCAGTATAAACCTGGTAAGTTGTTTCGGATCTCTGAGACCATTCGGTATTGATATCGTCCAGAGATTCGTAGAAACGAAGAAAGCTCTCAGTTTTAAGGCTTTCCTCAGGTTGTAGTTGGCTCTGATGTGGATCCTGAACCTCGGTCCGTATTGTCCCGGTGGGAAGATCTCGTATCCAGTTACGTGGAGGTATTCCTTGTCGCCAAAAATGTGATGCCAAAC

CGCGGTACGACTGAGCCAATGGAACTTGGTGTTGTAACTGACATCCATTGGTATGAAATAAGTGACACTGGCATTCGAACTTAATGAGTTTCTGTCGTTGGGATTTGGGACGTCGCTTTGCCTGACGATGTTGGACTTTGATGCTCGGTGGCATAGAGTGAGCTGTTGATGGTGATGAACTCCACATTCTGTATCGTCCATTCACGAAGTGCCTGATTTTCTGGTTTGTTGAGAAATTCCTTATAAGATGATCCTTGACCTGGATTGCATAAGACAATAGTTGGGATTCCACCCTTGATG

TGACATGGCTTGGCGTATTTACAATTTGAATTCCAATCTCGCTGCGCGCCGATGAACTCTTTCCAGTGCTTTAAGTACTGTGGATTGACGTCATCAATGACGTTAAAGTAACAATCATCATTGTGGTTCTTTAAATCAACGTGACCAGCATAATAATTGTGGGGACCCAGGCTTCTTGCCTAGCAAGTTTTACCACATCTAGATGGTCCTTCTAGAATCAGGCTGATGGGTCTGTGTGGTCTATGGGCTTCATCTCTGATGTTATCTGAAGCCCATTGTTGTAGGTCCACCAGAACATTA

TTTGATGGCGGAAAAGGATTTTGGTATGGTGGTTCTGGTTCCTTGAAAATATTACGGAGATTTGCAGAAAATCGATCGTAATTAAGAATAAAAGAAAATGGATCCCCTTCTCTAATAACCCTTAATGCATCATTTACTGAACCGGTGGCTAGCGCAGCGACAACAATGTCTTTCTTTGAAACAGACGTCTTACAACATTTGAACTGACCTTGATCTATGAAGTTGGAGTATTTTGAGATGTAATCTTTGACTGCCGATTCATTTCTAGCTCCCTGAACATTCGGATGGAAATGTGTTGAC

CTGGTTCTAGATACCAAGTCAAAGAAACGCTGATTTGTTGTTTGGAACTTGTAAGGGAATTGAAGTAGACAGTGGAGATGAGGTTCCCCATCTTCGTGTACTTCTGCGGCAACTCTGATGAAAAACGGTGGTCTTGAATTGAACAGAGCTTGAATCAGAGACAAAGCCTCCTCCTTTGGAAGTGGACATTTAGGATAAGTAAGAAAAACATTTTTCACACATAAACAGAACGAAGAAGAACGCGGCATATGGAATATTTTTACTAACACCAGCTAAAAGTATCTCCCACCCCGGTGTCAC

TGGCGTGAGTTATATAGGGGGTATTTGTGGTACCCCTAGAGCGCCCTTATAATATTACTAAGGGCGCTCCCGTGCCACGTGGACGGCCAGGATCAAAGATCCTAGACAGACACAACGGTTAGGATTTGTCGTAACGGCTTTTTCTCCACAAAACCCGGCGCAAAGGTGAACAGTGGTGGACCAATGACCAAAATACCCATGGTGTGAGTGGCATAATTTCGGGAATGCCATTGGCATAAGTTGTGAAGACCCCACCATCTCTTGGCTTCATCTCTGATGTTACCTAAAGCACATTGTGGT

TGTCATTGACGTGACATATTGTACTGCACTGGACAGATGGTCTTAGATCATTGGCTGGTTGTAAAGGGTTGCACCGATGACCTGTTTGAACACGATTGGTTGGTTTTGAACGTGGTATTGGACATTTGGTCGCAGGTGATTGTTTGTTGTTGTTATAAAAACAGATGTCACTCGTTTAACATATCACTAAGTTTCAGATCAGGTGTATTGGTATTGTTGGTCATATTGTTTCTCTGTAACCGTCCGATTTAATTGATCTTCATCAATTATGTAAGGAGTGAAGAGGAAGCAGATCATCCC

AGAGATATCTTCATCAATGATAATATTTATTATAAAGGATTTTGCACCACAAAGCCGGAGACAATGGTGAACAGAACTTAGATAATGAGTAAAATATGCCTCGAATGAGTTGTATAAATTCGGGAATGCCATTGGCTTAAGATGGTAAATATAAAATGATAGTGTGGAGTCACGCACCTCTGTGGATCATGGAGGATAAGGACTCTCTTATTTTGTTGCGGTTATTGAGTCGAAATGCTTTTAGGGTTATTCTCCGAGAATTAACATCTTGGTACAGTCATGACACGGAGTTCTTGATCA

TTTGGATCAATGGGCTGTAGGCAGAATAAAAGAACATTTTTCGAGCACTCATCTCTTTACTAGACATTGAAGTCTCA
