# Supplementary material for: Characterisation of novel endogenous geminiviral elements in macadamia
Source: BMC Genomics. 2021 Nov 27;22:858. doi: 10.1186/s12864-021-08174-0 (PMC8626973; doi:10.1186/s12864-021-08174-0)
Supplement: Supplementary file 2 — Additional file 2: Supplementary Figure 1. Full length gel images of Figs. 4a, b and 5a, b and c included in this article. [file 12864_2021_8174_MOESM2_ESM.pdf]

21112019 nodPCR

Fig 4(a)

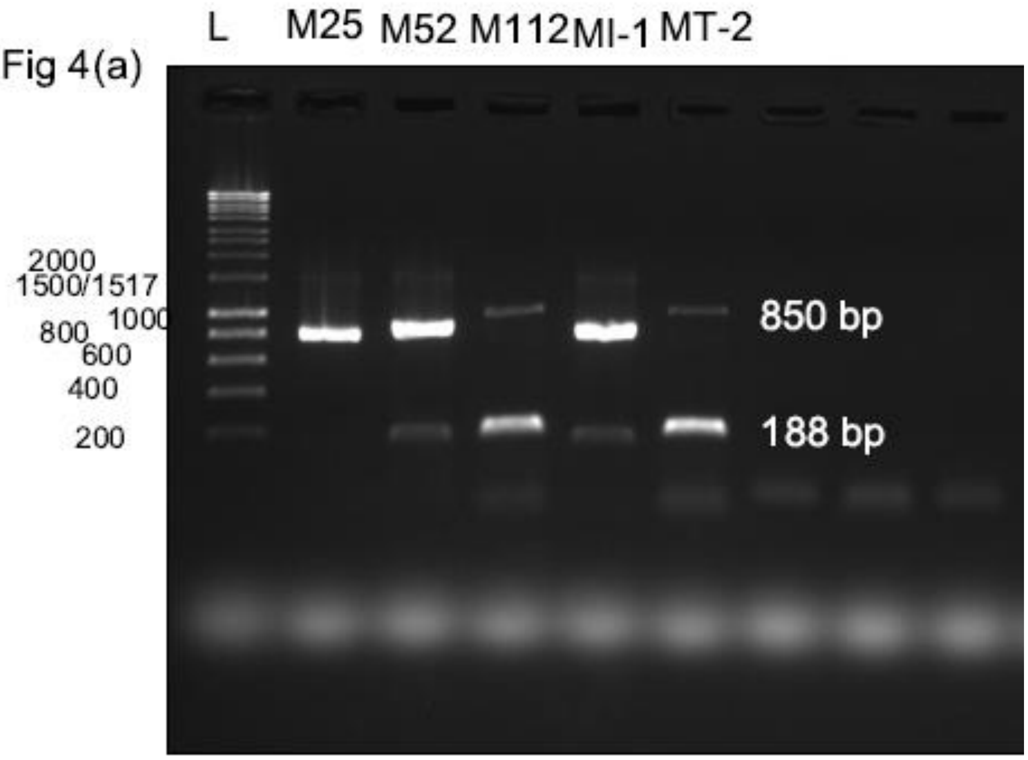

Location: C:/Users/Eliza Doolittle/Desktop/Zakeel
Printed: 21/11/2019 7:41 PM

05012020

Fig 4(b)

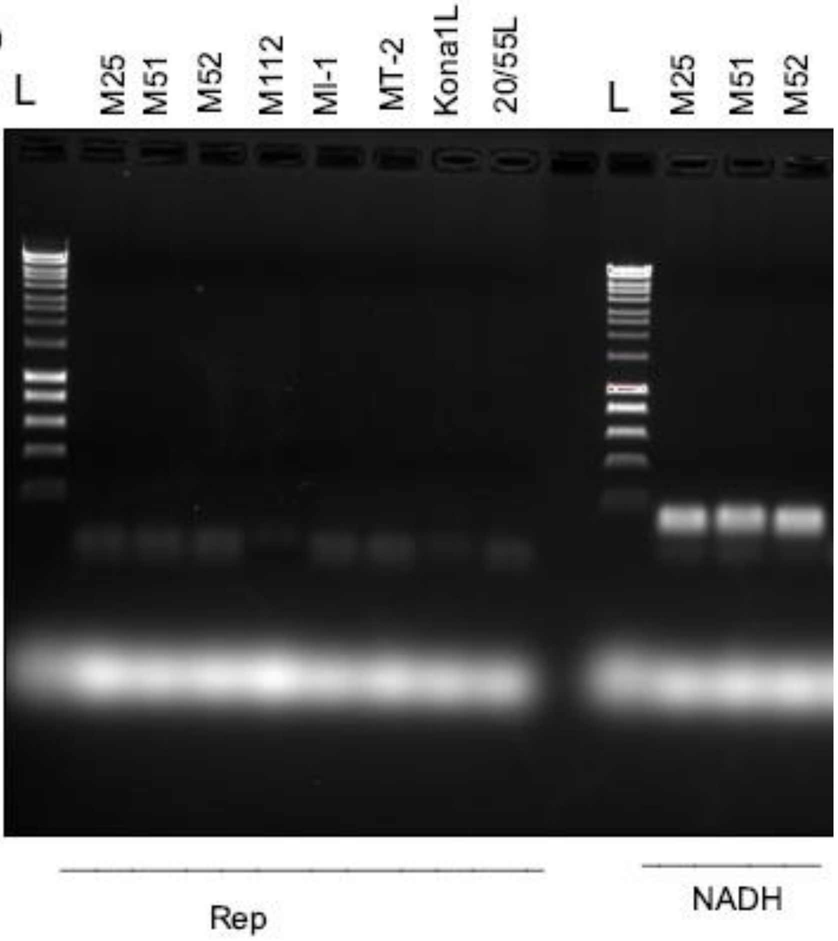

Location: C:/Users/Eliza Doolittle/Desktop/Zakeel
Printed: 5/01/2020 4:48 PM

Fig 5(a)

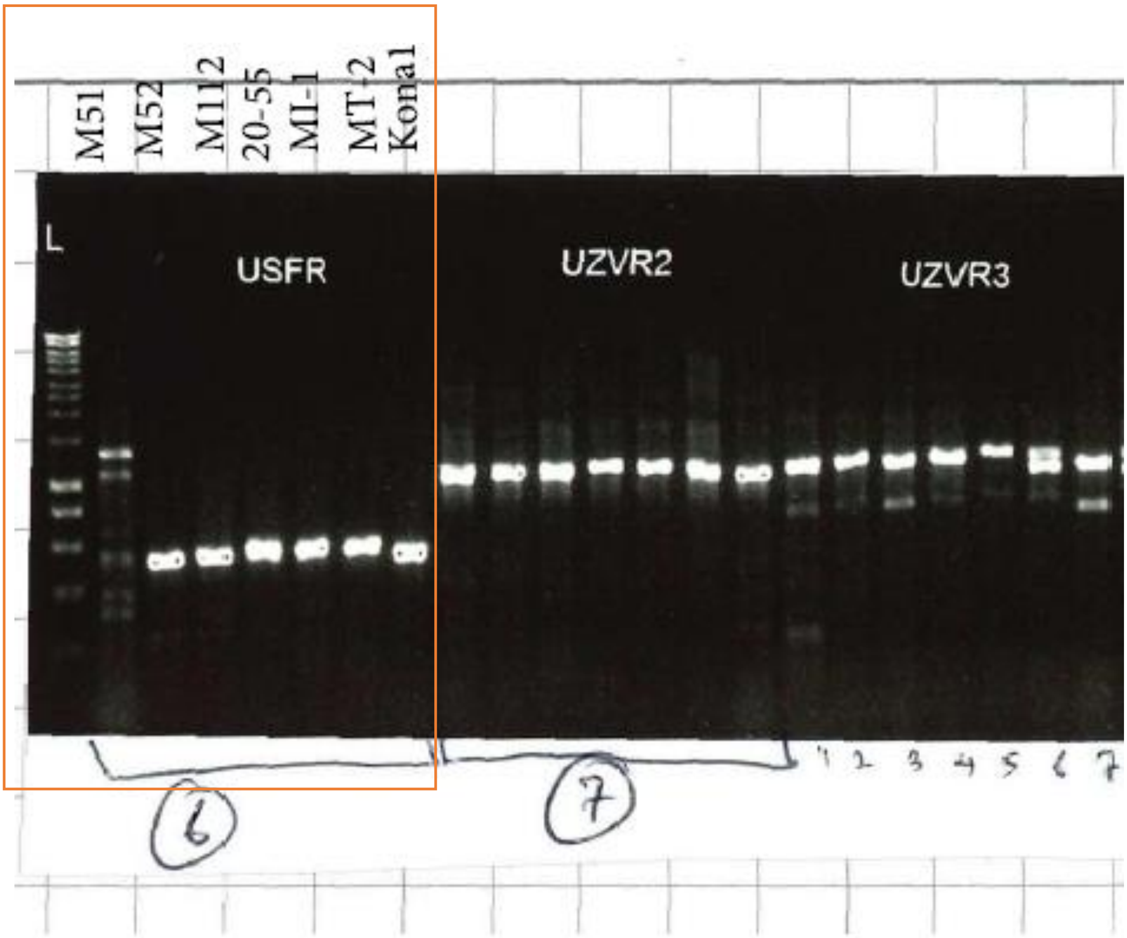

Fig 5(b)

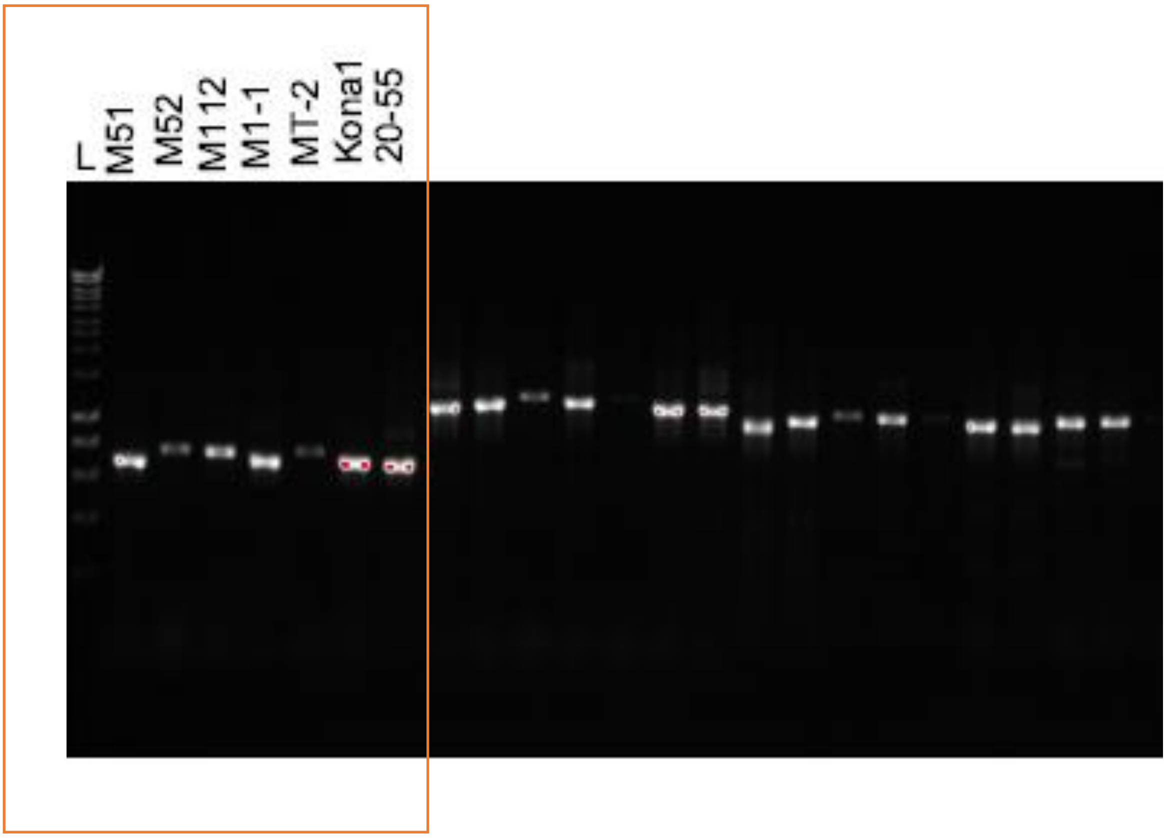

Location: C:/Users/Eliza Doolittle/Desktop/Zakeel
Printed: 17/09/2019 8:03 PM

Fig 5(c)

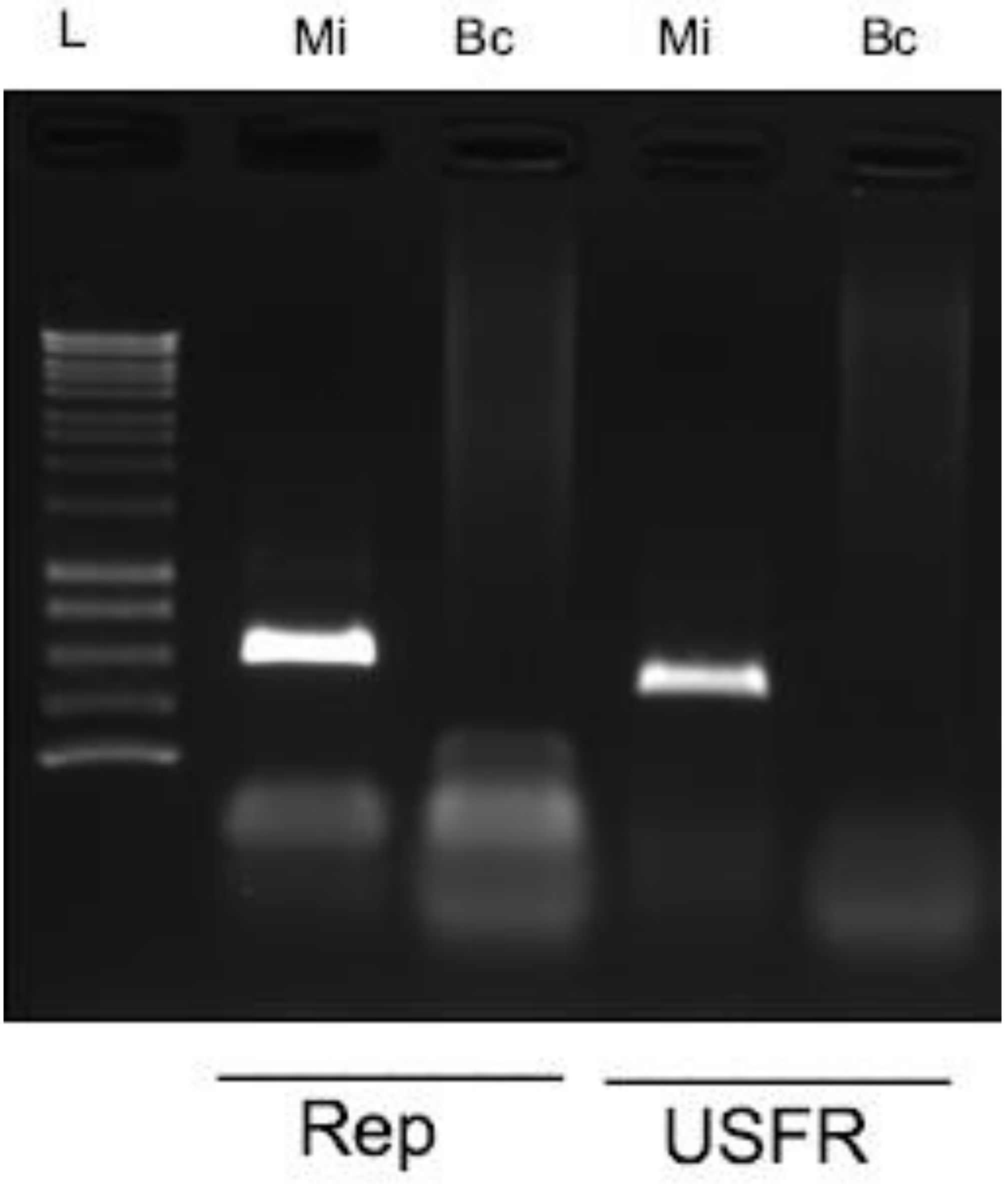

Location: C:/Users/Eliza Doolittle/Desktop/Zakeel1
Printed: 19/08/2020 6:11 PM
