# Supplementary figures and images for: Characterisation of novel endogenous geminiviral elements in macadamia
Source: BMC Genomics. 2021 Nov 27;22:858. doi: 10.1186/s12864-021-08174-0 (PMC8626973; doi:10.1186/s12864-021-08174-0)

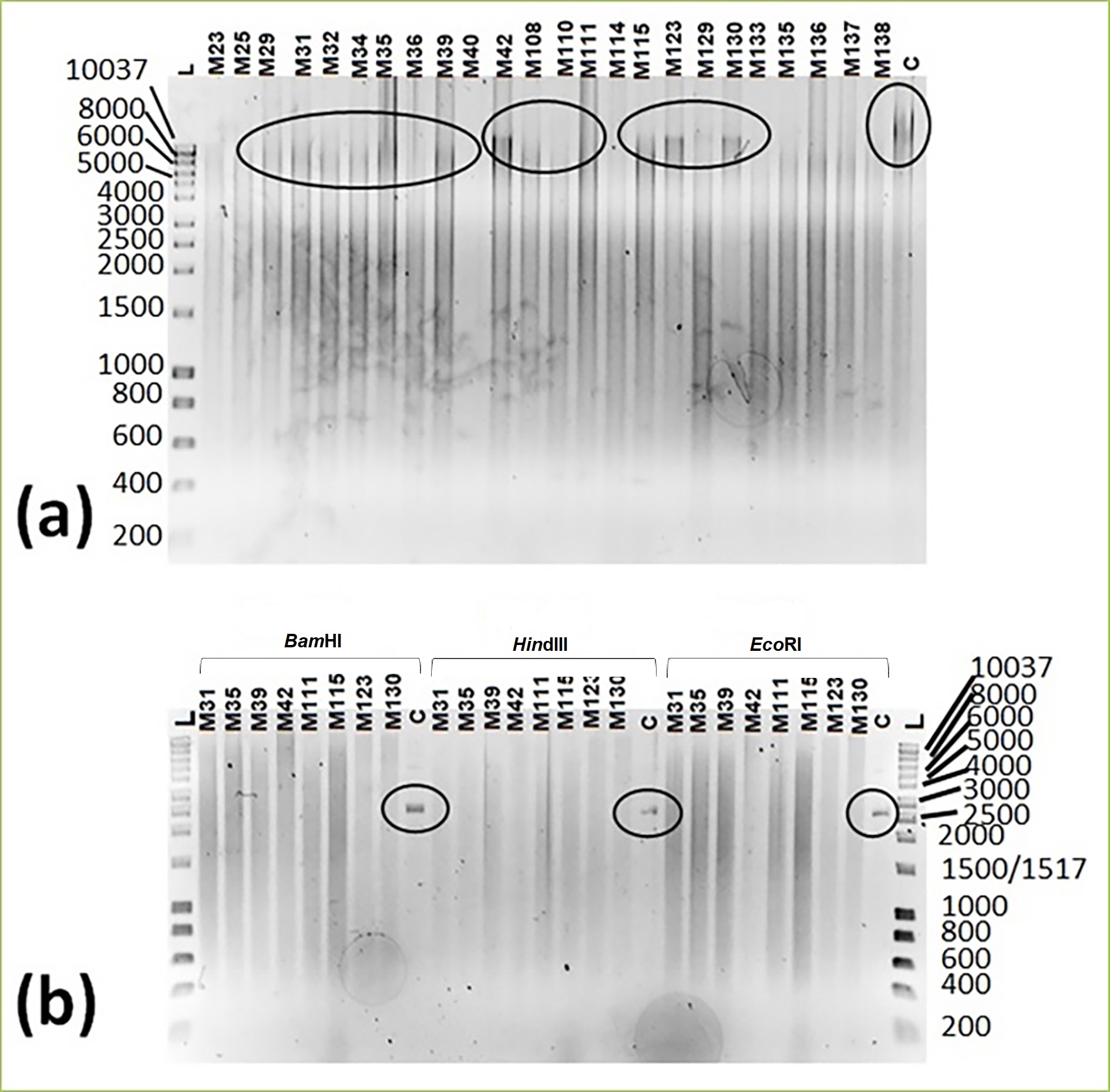

Supplement: Supplementary file 3 — Additional file 3: Supplementary Figure 2. Detection of circular forms of geminiviral DNA using the TempliPhi assay, a sequence non-specific amplification method. [file 12864_2021_8174_MOESM3_ESM.jpg]
